# Monitoring Parameter Change for Time Series Models of Counts Based on Minimum Density Power Divergence Estimator

**DOI:** 10.3390/e22111304

**Published:** 2020-11-16

**Authors:** Sangyeol Lee, Dongwon Kim

**Affiliations:** Department of Statistics, Seoul National University, Seoul 08826, Korea; dongwon.k@snu.ac.kr

**Keywords:** time series of counts, INGARCH model, SPC, CUSUM monitoring, MDPDE

## Abstract

In this study, we consider an online monitoring procedure to detect a parameter change for integer-valued generalized autoregressive heteroscedastic (INGARCH) models whose conditional density of present observations over past information follows one parameter exponential family distributions. For this purpose, we use the cumulative sum (CUSUM) of score functions deduced from the objective functions, constructed for the minimum power divergence estimator (MDPDE) that includes the maximum likelihood estimator (MLE), to diminish the influence of outliers. It is well-known that compared to the MLE, the MDPDE is robust against outliers with little loss of efficiency. This robustness property is properly inherited by the proposed monitoring procedure. A simulation study and real data analysis are conducted to affirm the validity of our method.

## 1. Introduction

In this paper we consider the cumulative sum (CUSUM) monitoring procedure for detecting a parameter change in integer-valued generalized autoregressive heteroscedastic (INGARCH) models. Integer-valued time series is a core area in time series analysis that includes diverse disciplines in social, physical, engineering, and medical sciences. Both integer-valued autoregressive (INAR) time series models and the integer-valued generalized autoregressive conditional heteroscedastic (INGARCH) models have been widely studied in the literature and applied to various practical problems. Refer to McKenzie [1], Al-Osh and Alzaid [2], Ferland, Latour and Oraichi [3], Fokianos, Rahbek and Tjøstheim [4], and Weiβ [5] for a general review. Poisson, negative binomial (NB), and one-parameter exponential family distributions have been widely used as underlying distributions, as seen in Davis and Wu [6], Zhu [7], Zhu [8], Jazi, Jones and Lai [9], Christou and Fokianos [10], Davis and Liu [11], Lee, Lee and Chen [12], and Chen, Khamthong and Lee [13].

Since Page [14], the CUSUM test has been a conventional tool to detect a structural change in underlying models. For a history and background, we refer to Csörgö and Horváth [15], Chen and Gupta [16], Lee, Ha, Na and Na [17], and the papers cited therein. Several authors have studied the change point test for INGARCH models, including Fokianos and Fried [18], Fokianos and Fried [19], Franke, Kirch and Kamgaing [20], Fokianos, Gombay and Hussein [21], Hudecová [22], Hudecová, HuŠková and Meintanis [23], Kang and Lee [24], Lee, Lee and Chen [12], Lee, Lee and Tjøstheim [25], and Lee and Lee [26]. This CUSUM scheme has been applied not only to retrospective change point tests but also to on-line monitoring and statistical process control (SPC) problems, designed to monitoring abnormal phenomena in manufacturing processes and health care surveillance. The CUSUM control chart has been popular due to its considerable competency in early detection of anomalies. Refer to Weiβ [27], Rakitzis, Maravelakis and Castagliola [28], Kim and Lee [29], and the papers cited therein. Meanwhile, Gombay and Serban [30] used the CUSUM approach based on the score vectors for independent observations, and later extended it to autoregressive processes, wherein the Type I probability error is measured for obtaining control limits instead of the conventional average run length (ARL). Their CUSUM monitoring process is based on the asymptotic property of the partial sum process generated from score vectors. Later, Huh, Kim and Lee [31] adopted their method for analyzing Poisson INGARCH models, and compared its performance with the likelihood ratio (LR)-based control chart, originally considered by Weiss and Testik [32].

In this work, taking the approach of Gombay and Serban [30] and Huh, Kim and Lee [31], we designate a robust monitoring process based on the minimum distance power divergence estimator (MDPDE) proposed by Basu, Harris, Hjort and Jones [33]. We do this because the MDPDE is well-known to be suitable for robust inference in various models, having a trade-off between efficiency and robustness controlled through the tuning parameters with little loss in asymptotic efficiency relative to the maximum likelihood estimator (MLE) (Riani, Atkinson, Corbellini and Perrotta [34]). The MDPDE method has been successfully applied to various time series models, and in particular INGARCH models (Kim and Lee [35], Kim and Lee [36]). Recently, Lee and Lee [26] and Kim and Lee [37] considered the CUSUM tests based on score vectors for the MLE and MDPDE in exponential family distribution INGARCH models. See also Kang and Song [38]. Using their results within the framework of Gombay and Serban [30] and Huh, Kim and Lee [31], we design an MDPDE-based monitoring process to detect a model parameter change in INGARCH models. Monte Carlo simulations are conducted to assess the performance of the proposed monitoring procedure. A focus is made on comparing the MDPDE-based CUSUM test with the MLE-based CUSUM test for Poisson INGARCH models to demonstrate the superiority of the former over the latter in the presence of outliers. A real data analysis of the return times of extreme events of Goldman Sachs Group (GS) stock prices is also provided to illustrate the validity of the proposed test.

The rest of the paper is organized as follows. Section 2 reviews the MDPDE for INGARCH models and Section 3 constructs the monitoring procedure for these models and investigates its asymptotic properties. Section 4 presents a simulation study and Section 5 provides a real data analysis. Section 6 concludes the paper. The proof of the main theorem is provided in Appendix A.

## 2. MDPDE for INGARCH Model: An Overview

In this section, we briefly review the MDPDE for INGARCH models in [36]. Let $Y_{1},Y_{2},\ldots$ be the observations generated from integer-valued time series models with the conditional distribution of the one-parameter exponential family:

(1) $$\begin{array}{c} Y_{t}|F_{t-1}∼p\left(y|\eta_{t}\right),\;\;\;X_{t}:=E\left(Y_{t}|F_{t-1}\right)=f_{\theta }\left(X_{t-1},Y_{t-1}\right), \end{array}$$

where $F_{t-1}$ is a σ-field generated by $Y_{t-1},Y_{t-2},\ldots$, and $f_{\theta }\left(x,y\right)$ is a non-negative bivariate function, depending on the parameter $\theta \in \Theta \subset R^{d}$, and satisfies $\inf_{\theta \in \Theta }f_{\theta }\left(x,y\right)\geq c_{*}$ for some $c_{*}>0$ for all x,y, and p(·|·) is a probability mass function given by

$$\begin{array}{c} p(y|\eta )=\exp\{\eta y-A(\eta )\}h(y),\;\;\;y=0,1,\ldots , \end{array}$$

where η is the natural parameter, A(η) and h(y) are known functions, and both *A* and $B=A^{{}^{\prime }}$ are strictly increasing. In particular, $B\left(\eta_{t}\right)=X_{t}$ and $B^{\prime }\left(\eta_{t}\right)$ is the conditional variance of $Y_{t}$. In what follows, symbols $X_{t}\left(\theta \right)$ and $\eta_{t}\left(\theta \right)=B^{-1}\left(X_{t}\left(\theta \right)\right)$ are also utilized to stand for $X_{t}$ and $\eta_{t}$, respectively.

Davis and Liu [11] demonstrated that the strict stationarity and ergodicity of $\left\{X_{t}\right\}$, and the expression of $X_{t}\left(\theta \right)=f_{\infty }^{\theta }\left(Y_{t-1},Y_{t-2},\ldots \right)$ are allowed for some nonnegative measurable function $f_{\infty }^{\theta }$ defined on $N_{0}^{\infty }$ under the contraction condition: for all $x,x^{\prime }\geq 0$ and $y,y^{\prime }\in N_{0}$,

$$\begin{array}{c} \underset{\theta \in \Theta }{\sup}|f_{\theta }\left(x,y\right)-f_{\theta }\left(x^{\prime },y^{\prime }\right)|\leq \lambda_{1}|x-x^{\prime }|+\lambda_{2}\left|y-y^{\prime }\right| \end{array}$$

with constants $\lambda_{1},\lambda_{2}\geq 0$ satisfying $\lambda_{1}+\lambda_{2}<1$.

Meanwhile, Basu, Harris, Hjort and Jones [33] considered the minimum distance power divergence estimator (MDPDE) for model parameters using the density power divergence $d_{\alpha }$ between two density functions *g* and *h*, defined by:

$$d_{\alpha }\left(g,h\right):=\left\{\begin{array}{cc} \int \{g^{1+\alpha }\left(y\right)-\left(1+\frac{1}{\alpha }\right)h\left(y\right)g^{\alpha }\left(y\right)+\frac{1}{\alpha }h^{1+\alpha }\left(y\right)\}dy, & \alpha >0,\\ \int h(y)(\log h(y)-\log g(y))dy, & \alpha =0. \end{array}\right.$$

Kim and Lee [36] studied the MDPDE for one parameter exponential family distribution INGARCH models. Given $Y_{1},\ldots ,Y_{n}$ generated from (1), the MDPDE is defined by

(2) $$\begin{array}{c} {\hat{\theta }}_{\alpha ,n}=\underset{\theta \in \Theta }{\mathrm{argmin}}{\tilde{L}}_{\alpha ,n}\left(\theta \right)=\underset{\theta \in \Theta }{\mathrm{argmin}}\frac{1}{n}\sum_{t=1}^{n}{\tilde{l}}_{\alpha ,t}\left(\theta \right), \end{array}$$

where

(3) $$\begin{array}{cc} {\tilde{l}}_{\alpha ,t}\left(\theta \right) & =\left\{\begin{array}{cc} \sum_{y=0}^{\infty }p^{1+\alpha }\left(y|{\tilde{\eta }}_{t}\left(\theta \right)\right)-\left(1+\frac{1}{\alpha }\right)p^{\alpha }\left(Y_{t}|{\tilde{\eta }}_{t}\left(\theta \right)\right), & \alpha >0,\\ -\log p(Y_{t}|{\tilde{\eta }}_{t}\left(\theta \right)), & \alpha =0, \end{array}\right. \end{array}$$

and ${\tilde{\eta }}_{t}\left(\theta \right)=B^{-1}\left({\tilde{X}}_{t}\left(\theta \right)\right)$ is updated recursively through the equations: ${\tilde{X}}_{t}\left(\theta \right)=f_{\theta }\left({\tilde{X}}_{t-1}\left(\theta \right),Y_{t-1}\right),\;t\geq 2$ with an initial value ${\tilde{X}}_{1}\left(\theta \right):={\tilde{X}}_{1}$.

Below, $\theta_{0}$ denotes the true value of θ and is assumed to be an interior point in the compact parameter space $\Theta \subset R^{d}$. Moreover, it is assumed that $E{\left(\sup_{\theta \in \Theta }X_{1}\left(\theta \right)\right)}^{4}<\infty$, $EY_{1}^{4}<\infty$, $X_{t}\left(\theta \right)=X_{t}\left(\theta_{0}\right)$ a.s. implies $\theta =\theta_{0}$, and $\nu^{T}\frac{\partial X_{t}\left(\theta_{0}\right)}{\partial \theta }=0$ a.s. implies ν=0. Furthermore, $\theta \mapsto X_{t}\left(\theta \right)$ is twice continuously differentiable with respect to θ and satisfies

$$\begin{array}{c} E{\left(\underset{\theta \in \Theta }{\sup}∥\frac{\partial X_{t}\left(\theta \right)}{\partial \theta }∥\right)}^{4}<\infty \;\;\;\;\;\mathrm{and}\;\;\;E{\left(\underset{\theta \in \Theta }{\sup}∥\frac{\partial^{2}X_{t}\left(\theta \right)}{\partial \theta \partial \theta^{T}}∥\right)}^{2}<\infty . \end{array}$$

Assuming

$$\underset{\theta \in \Theta }{\inf}\underset{0\leq \delta \leq 1}{\inf}B^{\prime }\left(\left(1-\delta \right)\eta_{t}\left(\theta \right)+\delta {\tilde{\eta }}_{t}\left(\theta \right)\right)\geq \underline{c}$$

for some $\underline{c}>0$, Kim and Lee [36] verified that the MDPDE is strongly consistent. Additionally, they showed that provided

$$\underset{\theta \in \Theta }{\sup}\underset{0\leq \delta \leq 1}{\sup}\{\left|\frac{B^{\prime \prime }\left(\left(1-\delta \right)\eta_{t}\left(\theta \right)+\delta {\tilde{\eta }}_{t}\left(\theta \right)\right)}{B^{\prime }{\left(\left(1-\delta \right)\eta_{t}\left(\theta \right)+\delta {\tilde{\eta }}_{t}\left(\theta \right)\right)}^{5/2}}\right|\leq K\;\;\mathrm{for}\;\mathrm{some}\;K>0,$$

and

$$\underset{\theta \in \Theta }{\sup}∥\frac{\partial {\tilde{X}}_{t}\left(\theta \right)}{\partial \theta }-\frac{\partial X_{t}\left(\theta \right)}{\partial \theta }∥+∥\frac{\partial^{2}{\tilde{X}}_{t}\left(\theta \right)}{\partial \theta \partial \theta^{T}}-\frac{\partial^{2}X_{t}\left(\theta \right)}{\partial \theta \partial \theta^{T}}∥\leq V\rho^{t}\;a.s.,$$

where *V* and ρ∈(0,1) denote a generic integrable random variable and a constant, respectively, the symbol ∥·∥ denotes the $L^{2}$-norm for matrices and vectors, and expectation E(·) is taken under $\theta_{0}$, the MDPDE is asymptotically normal with asymptotic variance $J_{\alpha }^{-1}K_{\alpha }J_{\alpha }^{-1}$ where

(4) $$\begin{array}{c} J_{\alpha }=-E\left(\frac{\partial^{2}l_{\alpha ,t}\left(\theta_{0}\right)}{\partial \theta \partial \theta^{T}}\right),\;K_{\alpha }=E\left(\frac{\partial l_{\alpha ,t}\left(\theta_{0}\right)}{\partial \theta }\frac{\partial l_{\alpha ,t}\left(\theta_{0}\right)}{\partial \theta^{T}}\right), \end{array}$$

and $l_{\alpha ,t}\left(\theta \right)$ is the same as ${\tilde{l}}_{\alpha ,t}\left(\theta \right)$ with ${\tilde{\eta }}_{t}\left(\theta \right)$ in (3) replaced by $\eta_{t}\left(\theta \right)$.

Moreover, additionally assuming

$$\underset{\theta \in \Theta }{\sup}\underset{0\leq \delta \leq 1}{\sup}\left|\frac{B^{\left(3\right)}\left(\left(1-\delta \right)\eta_{t}\left(\theta \right)+\delta {\tilde{\eta }}_{t}\left(\theta \right)\right)}{B^{\prime }{\left(\left(1-\delta \right)\eta_{t}\left(\theta \right)+\delta {\tilde{\eta }}_{t}\left(\theta \right)\right)}^{4}}\right|\leq M\;\;\mathrm{for}\;\mathrm{some}\;M>0,$$

Kim and Lee [37] showed that the CUSUM test statistics designed for detecting a change in θ have the limiting null distribution of the sup of a Brownian bridge. In practice, α∈(0,1] is often harnessed and an optimal α can be selected through the method of Warwick [39] and Warwick and Jones [40]; see Remark 1 of Kim and Lee [36].

In the literature, the following linear INGARCH model has been frequently used:

$$\begin{array}{c} Y_{t}|F_{t-1}∼p\left(y|\eta_{t}\right),\;\;\;X_{t}=\omega +aX_{t-1}+bY_{t-1}, \end{array}$$

where $X_{t}=B\left(\eta_{t}\right)=E\left(Y_{t}|F_{t-1}\right)$ and $\theta ={\left(\omega ,a,b\right)}^{T}$ satisfy ω>0 and a+b<1. Here, we assume that $\theta_{0}$ is an interior of a compact neighborhood $\Theta =\left\{\theta ={\left(\omega ,a,b\right)}^{T}\in R_{+}^{3}:0<\omega_{1}\leq \omega \leq \omega_{2},\;\epsilon \leq a+b\leq 1-\epsilon \right\}$ for some $0<\omega_{1}<\omega_{2},\;\epsilon >0.$ Moreover, the Poisson INGARCH(1,1) model with $Y_{t}|F_{t-1}∼\mathrm{Poisson}\left(X_{t}\right)$ and the NB-INGARCH(1,1) model with $Y_{t}|F_{t-1}∼\mathrm{NB}\left(r,p_{t}\right),\;\;\;X_{t}=\frac{r(1-p_{t})}{p_{t}}$, where NB(r,p) denotes a negative binomial (NB) distribution with parameters r∈N and p∈(0,1), satisfy the aforementioned regularity conditions. Those conditions should be checked analytically when one aims to use a specific distribution as the conditional distribution of the INGARCH model. In this case, a goodness of fit test could be conducted to check the adequacy of the assumed underlying distribution (Fokianos and Neumann [41]).

## 3. MDPDE-Based Monitoring Process

In this section, we consider a monitoring process detecting a significant change in the underlying models based on sequentially observed time series $Y_{1},\ldots ,Y_{n}$ following Model (1), given a training sample $Y_{1}^{{}^{\prime }},\ldots ,Y_{m}^{{}^{\prime }}$ from Model (1), where m=m(n) is a sequence of positive integers that diverges to *∞* as *n* tends to *∞*. For this task, we set up the following hypotheses:

$$H_{0}:\theta \;\;\;\mathrm{does}\;\mathrm{not}\;\mathrm{change}\;\mathrm{over}\;t=1,\ldots ,n\;\;\;vs.\;\;\;H_{1}:\;\mathrm{not}\;\;H_{0}.$$

We first consider the case that $\theta_{0}$ is known a priori from a past experience. Then we consider the monitoring process using the process ${\hat{W}}_{k,0}={\hat{K}}_{\alpha }^{-1/2}\sum_{t=1}^{k}\frac{\partial {\tilde{l}}_{\alpha ,t}\left(\theta_{0}\right)}{\partial \theta }$, k=1,…,n, constructed as

(5) $$\begin{array}{ccc} & & {\hat{T}}_{n,0}^{min}:=\max_{1\leq k\leq n}{\hat{T}}_{n,0}^{min}\left(k\right)=\max_{1\leq k\leq n}\frac{1}{\sqrt{n}}{\left|\left|\min_{j\leq k}{\hat{W}}_{j,0}-{\hat{W}}_{k,0}\right|\right|}_{\max},\\ & & {\hat{T}}_{n,0}^{max}:=\max_{1\leq k\leq n}{\hat{T}}_{n,0}^{max}\left(k\right)=\max_{1\leq k\leq n}\frac{1}{\sqrt{n}}{\left|\left|\max_{j\leq k}{\hat{W}}_{j,0}-{\hat{W}}_{k,0}\right|\right|}_{\max},\\ & & {\hat{T}}_{n,0}^{cusum}:=\max_{1\leq k\leq n}{\hat{T}}_{n,0}^{{}^{\prime }}\left(k\right)=\max_{1\leq k\leq n}\max_{1\leq i<j\leq k}\frac{1}{\sqrt{n}}\left|\left|\left(\frac{i}{j}\right){\hat{W}}_{j,0}-{\hat{W}}_{i,0}\right|\right|, \end{array}$$

where $\frac{\partial {\tilde{l}}_{\alpha ,t}}{\partial \theta }$ is the score vector as in (3) based on $Y_{1},\ldots ,Y_{n}$ and

(6) $$\begin{array}{c} {\hat{K}}_{\alpha }=\frac{1}{m}\sum_{t=1}^{m}\frac{\partial {\tilde{l}}_{\alpha ,t}^{{}^{\prime }}\left(\theta_{0}\right)}{\partial \theta^{T}}\frac{\partial {\tilde{l}}_{\alpha ,t}^{{}^{\prime }}\left(\theta_{0}\right)}{\partial \theta^{T}}, \end{array}$$

where $\frac{\partial {\tilde{l}}_{\alpha ,t}^{{}^{\prime }}}{\partial \theta }$ is the score vector based on the training sample. Here, the notation $\max_{1\leq i\leq k}z_{i}$ with $z_{i}={\left(z_{i,1},\ldots ,z_{i,d}\right)}^{T}\in R^{d}$ is defined to be the vector with the *j*th entry equal to $\max_{1\leq i\leq k}z_{j,i}$ for j=1,…,d, and ${\left||z|\right|}_{\max}=\max_{1\leq i\leq k}\left|z_{i}\right|$ for $z={\left(z_{1},\ldots ,z_{d}\right)}^{T}\in R^{d}$. Similar versions of ${\hat{T}}_{n,0}^{max}$ and ${\hat{T}}_{n,0}^{cusum}$ based on MLE have been considered by Gombay and Serban [30] and Huh, Kim and Lee [31] for the AR and Poisson INGARCH models, while ${\hat{T}}_{n,0}^{min}$ is newly considered here. An anomaly is signaled at *k* when ${\hat{T}}_{n,0}^{min}\left(k\right)$, ${\hat{T}}_{n,0}^{max}\left(k\right)$, or ${\hat{T}}_{n,0}^{cusum}\left(k\right)$ get out of a control limit for some k=1,…,n, and the control limit can be determined using the convergence result in Theorem 1 addressed below.

Next, we consider the situation that $\theta_{0}$ is unknown and must be estimated in the construction of the monitoring process in (5). We employ a monitoring process constructed based on ${\hat{W}}_{k}={\hat{K}}_{\alpha ,m}^{-1/2}\sum_{t=1}^{k}\frac{\partial {\tilde{l}}_{\alpha ,t}\left({\hat{\theta }}_{\alpha ,m}\right)}{\partial \theta }$, where ${\hat{\theta }}_{\alpha ,m}$ is the MDPDE of $\theta_{0}$ obtained from the training sample and

$${\hat{K}}_{\alpha ,m}=\frac{1}{m}\sum_{t=1}^{m}\frac{\partial {\tilde{l}}_{\alpha ,t}^{{}^{\prime }}\left({\hat{\theta }}_{\alpha ,m}\right)}{\partial \theta }\frac{\partial {\tilde{l}}_{\alpha ,t}^{{}^{\prime }}\left({\hat{\theta }}_{\alpha ,m}\right)}{\partial \theta^{T}},$$

which is obtained by substituting $\theta_{0}$ in $K_{\alpha }$ in (6) with ${\hat{\theta }}_{\alpha ,m}$, namely,

(7) $$\begin{array}{ccc} & & {\hat{T}}_{n}^{min}:=\max_{1\leq k\leq n}{\hat{T}}_{n}^{min}\left(k\right)=\max_{1\leq k\leq n}\frac{1}{\sqrt{n}}{\left|\left|\min_{j\leq k}{\hat{W}}_{j}-{\hat{W}}_{k}\right|\right|}_{\max},\\ & & {\hat{T}}_{n}^{max}:=\max_{1\leq k\leq n}{\hat{T}}_{n}^{max}\left(k\right)=\max_{1\leq k\leq n}\frac{1}{\sqrt{n}}{\left|\left|\max_{j\leq k}{\hat{W}}_{j}-{\hat{W}}_{k}\right|\right|}_{\max},\\ & & {\hat{T}}_{n}^{cusum}:=\max_{1\leq k\leq n}{\hat{T}}_{n}^{cusum}\left(k\right)=\max_{1\leq k\leq n}\max_{1\leq i<j\leq k}\frac{1}{\sqrt{n}}\left|\left|\left(\frac{i}{j}\right){\hat{W}}_{j,0}-{\hat{W}}_{i,0}\right|\right|. \end{array}$$

An anomaly is detected at *k* when ${\hat{T}}_{n}^{min}\left(k\right)$, ${\hat{T}}_{n}^{max}\left(k\right)$, or ${\hat{T}}_{n}^{cusum}\left(k\right)$ get out of the control limit for some k=1,…,n. The control limit can be determined theoretically using the asymptotic result in Theorem 1 addressed below. For this task, we investigate the asymptotic behavior of the monitoring processes ${\hat{T}}_{n}^{min},{\hat{T}}_{n}^{max}$, and ${\hat{T}}_{n}^{cusum}$ defined below.

Let $W_{k}=K_{\alpha }^{-1/2}\sum_{t=1}^{k}\frac{\partial l_{\alpha ,t}\left(\theta_{0}\right)}{\partial \theta }$, where $K_{\alpha }$ and $\frac{\partial l_{\alpha ,t}}{\partial \theta }$ are the ones in (4), and

$$\begin{array}{ccc} & & T_{n}^{min}=\max_{1\leq k\leq n}\frac{1}{\sqrt{n}}{\left|\left|\min_{j\leq k}W_{j}-W_{k}\right|\right|}_{\max},\\ & & T_{n}^{max}=\max_{1\leq k\leq n}\frac{1}{\sqrt{n}}{\left|\left|\max_{j\leq k}W_{j}-W_{k}\right|\right|}_{\max},\\ & & T_{n}^{cusum}=\max_{1\leq k\leq n}\max_{1\leq i<j\leq k}\frac{1}{\sqrt{n}}\left|\left|\left(\frac{i}{j}\right)W_{j}-W_{i}\right|\right|. \end{array}$$

Using Donsker’s invariance principle for martingale differences (Billingsley [42]) and the fact that $\sup_{0\leq s\leq t}B\left(s\right)-B\left(t\right)=\left|B\left(t\right)\right|$ in distribution for any standard Brownian motion *B*, we obtain

(8) $$\begin{array}{ccc} & & T_{n}^{max}\overset{d}{\to }T:=\underset{0\leq s\leq 1}{\sup}\left|\right|B_{d}\left(s\right){\left|\right|}_{\max}, \end{array}$$

where $B_{d}$ and denote a *d*-dimensional standard Brownian motion, so that

$$\begin{array}{c} T_{n}^{min}\overset{d}{\to }T=\underset{0\leq s\leq 1}{\sup}\left|\right|B_{d}\left(s\right){\left|\right|}_{\max} \end{array}$$

as $T_{n}^{min}$ behaves asymptotically similarly to $T_{n}^{max}$. Meanwhile, we can see that

(9) $$\begin{array}{c} T_{n}^{cusum}\overset{d}{\to }T^{{}^{\prime }}=\underset{0<s\leq s^{{}^{\prime }}\leq 1}{\sup}||\frac{s}{s^{{}^{\prime }}}B_{d}^{\circ }\left(s^{{}^{\prime }}\right)-B_{d}^{\circ }\left(s\right)||, \end{array}$$

where $B_{d}^{\circ }$ is a *d*-dimensional Brownian bridge.

Using the above facts, we are led to attain the following theorem, whose proof is provided in the Appendix A.

**Theorem** **1.** *Assume that* **(A.1)** *–* **(A.11)** *hold. Then, under $H_{0}$, as n→∞, ${\hat{T}}_{n,0}^{min}$ and ${\hat{T}}_{n,0}^{max}$ converge to T in distribution, and the same holds for ${\hat{T}}_{n}^{min}$ and ${\hat{T}}_{n}^{max}$ if m/n→∞. Moreover, ${\hat{T}}_{n,0}^{cusum}$ converges to $T^{{}^{\prime }}$ in distribution as n→∞, and so does ${\hat{T}}_{n}^{cusum}$ if m/n→λ∈(0,∞).*

The result in Theorem 1 can be used to determine a control limit for the monitoring process. Given significance level 0<α<1, we take *c* and $c^{{}^{\prime }}$ satisfying $P\left(T\geq c\right)=P\left(T^{{}^{\prime }}\geq c^{{}^{\prime }}\right)=\alpha$. In particular, $P\left(T\geq c\right)=1-(P(\sup_{0\leq s\leq 1}\left|B\left(s\right)\right|\leq {c))}^{d}$, so that *c* can be obtained from the fact that $P(\sup_{0\leq s\leq 1}{|B\left(s\right)|\geq c)=1-\left(1-\alpha \right)}^{1/d}$. The performance of the proposed CUSUM monitoring methods is evaluated in our simulation study, focusing on ${\hat{T}}_{n}^{cusum}$, ${\hat{T}}_{n,0}^{min}$, and ${\hat{T}}_{n}^{min}$. (We do not report the result for ${\hat{T}}_{n,0}^{max}$ and ${\hat{T}}_{n}^{max}$, as these do not perform well compared to the others in most cases). Therein, a parametric bootstrap is adopted in obtaining control limits to reduce the parameter estimation effect, which can be more problematic when *m* is not so large compared to *n*, and the MDPDE from the training sample is used to generate the bootstrap sample.

## 4. Simulation Results

In this section, we compare the performance of the CUSUM monitoring processes ${\hat{T}}_{n}^{cusum}$, ${\hat{T}}_{n,0}^{min}$, and ${\hat{T}}_{n}^{min}$ in three different experimental environments for the Poisson INGARCH(1,1) model as follows:

$$Y_{t}|F_{t-1}∼\mathrm{Poisson}\left(X_{t}\right),\;X_{t}=\omega +aX_{t-1}+bY_{t-1}.$$

For the comparison, we compute the empirical sizes and powers at the nominal level of 0.05 for m=n=500,1000 with 1000 implications. For the critical value of ${\hat{T}}_{n,0}^{min}$, we use 2.633, which is the 0.95th quantile of $\sup_{0\leq s\leq 1}{∥B_{3}\left(s\right)∥}_{\max}$. However, for ${\hat{T}}_{n}^{cusum}$ and ${\hat{T}}_{n}^{min}$, we use the critical values obtained from a parametric bootstrap method, as the MDPDE ${\hat{\theta }}_{\alpha ,m}$ might cause some size distortions. In implementation, the warp-bootstrap method is utilized to save computing times (Giacomini, Politis, and White [43]).

-Part 1. We compare the performance of MLE- and MDPDE-based monitoring processes (α=0,0.1,0.2,0.3) by calculating the size and power for the four different cases of changing parameter from $\left(\omega_{0},a_{0},b_{0}\right)$ to $\left(\omega_{1},a_{1},b_{1}\right)$ when the parameter change is assumed to occur at [n/2].

Case 1: $\omega_{1}=\left(1+\delta \right)\omega_{0}$, $a_{1}=\left(1+\delta \right)a_{0}$, $b_{1}=\left(1+\delta \right)b_{0}$; that is, all parameters change;

Case 2: $\omega_{1}=\left(1+\delta \right)\omega_{0}$, $a_{1}=a_{0}$, $b_{1}=b_{0}$; that is, only ω changes;

Case 3: $\omega_{1}=\omega_{0}$, $a_{1}=\left(1+\delta \right)a_{0}$, $b_{1}=b_{0}$; that is, only *a* changes;

Case 4: $\omega_{1}=\omega_{0}$, $a_{1}=a_{0}$, $b_{1}=\left(1+\delta \right)b_{0}$; that is, only *b* changes.

-Part 2. We examine the size and power for the same settings as in Part 1 when the change occurs at [n/4].

-Part 3. We compare the performance of MLE- and MDPDE-based monitoring processes (α=0,0.1,0.2,0.3) for the same settings as in Part 1 when outliers exist in the time series, wherein the parameter change is assumed to occur at [n/2]. In this case time series samples are generated from $\left(1-p_{t}\right)Y_{t}+p_{t}Z_{t}$ where $Y_{t}$ is the INGARCH process with the parameters as in Part 1, $p_{t}$ are iid Bernoulli random variables with success probability *p*, and $Z_{t}$ are iid Poisson variables wit intensity λ>0. Here, $\left\{Y_{t}\right\}$, $\left\{p_{t}\right\}$ and $\left\{Z_{t}\right\}$ are all independent.

**Figure 1:**
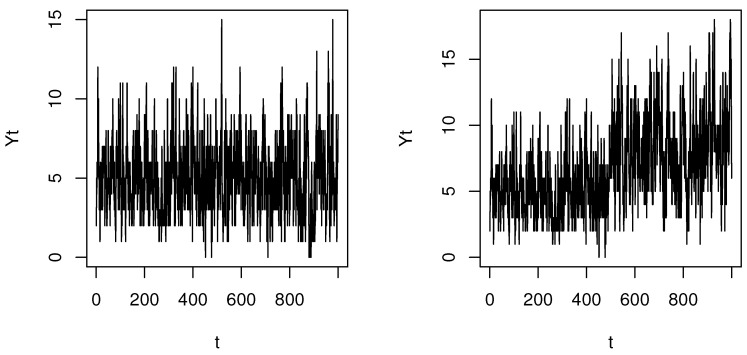
Plots of the Poisson INGARCH(1,1) time series (Case 3) with $\theta_{0}=\left(2,0.3,0.3\right)$, τ=500 and δ=0 for the left panel and δ=0.5 for the right panel.

Figure 1 shows how the parameter change affects the pattern of the Poisson INGARCH(1,1) time series (Case 3) with $\theta_{0}=\left(2,0.3,0.3\right)$, τ=500, and δ=0 for the left panel and δ=0.5 for the right panel. As $EY_{t}=\frac{\omega }{1-a-b}$, we can see that parameter change causes a mean shift. Table 1, Table 2 and Table 3 list the size and powers for Part 1 (τ therein stands for the location of the change point) and show no severe size distortions and reasonably good powers for δ≥0.5. In particular, ${\hat{T}}_{n}^{cusum}$ and ${\hat{T}}_{n,0}^{min}$ largely outperform ${\hat{T}}_{n}^{min}$ in terms of power. However, as seen in Table 4, Table 5, Table 6, Table 7 and Table 8, the power of ${\hat{T}}_{n}^{min}$ in Part 2 appears to increase up to that of ${\hat{T}}_{n,0}^{min}$. In both Part 1 and Part 2, different α do not affect the size much, but a larger α tends to diminish the power. This appeals to our intuition, as the MLE is more efficient in the presence of no outliers.

**Figure 2:**
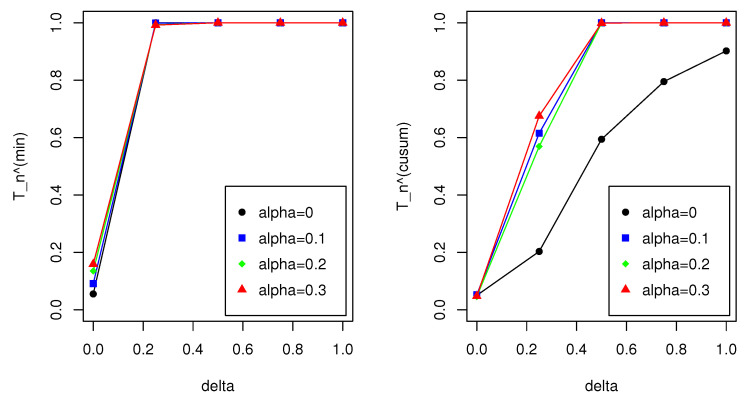
Plots of the sizes and powers in Table 10 (Part 3, Case 2) for n=1000. The left panel is for ${\hat{T}}_{n}^{min}$ and the right panel is for ${\hat{T}}_{n}^{cusum}$.

Meanwhile, Table 9, Table 10, Table 11 and Table 12 show that the outliers undermine the performance of the MLE-based monitoring processes in terms of both size and power; namely, size distortions are notable and the power decreases to a certain extent. This result particularly indicates that ${\hat{T}}_{n}^{cusum}$ is improved when the MDPDE with α>0 is used, which demonstrates the efficacy of the MDPDE in the monitoring process. By contrast, the size of ${\hat{T}}_{n}^{min}$ significantly increases when α>0, indicating that ${\hat{T}}_{n}^{min}$ is unstable; see Figure 2. Although not reported here, we also examined the performance of the same monitoring processes for NB INGARCH(1,1) models. The result for this case showed a similar pattern to the Poisson INGARCH(1,1) case. All our findings strongly affirm that ${\hat{T}}_{n}^{cusum}$ is the most favorable among the monitoring methods considered in this study.

## 5. Real Data Analysis

**Figure 3:**
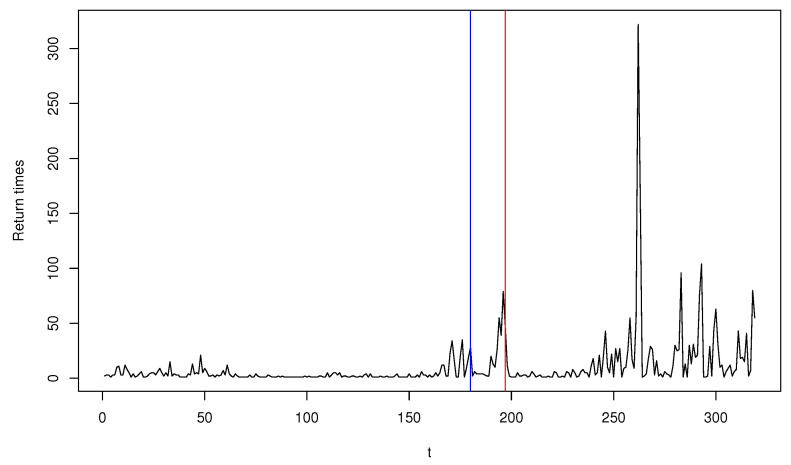
Plot of the return times of extreme events for Goldman Sachs Group stock.

In this section, we apply ${\hat{T}}_{n}^{cusum}$ to a real dataset, using the extreme events of the daily log-returns of GS stock from 2 July 2007 to 28 February 2020. Davis and Liu [11] and Kim and Lee [37] used the GS stock datasets with different periods, but their works were focused on parameter estimation and the retrospective change point test. For the task of online monitoring, we first calculated the hitting times, $\tau_{1},\tau_{2},\cdots ,$ for which the log-returns of GS stock fall outside the 0.05 and 0.95 quantiles of the data, and generated the time series of counts $Y_{t}=\tau_{t}-\tau_{t-1}\geq 0$, t=1,⋯,319. Figure 3 plots $Y_{t}$ and exhibits the presence of a number of outliers. Fitting the Poisson INGARCH(1,1) model to the whole observations, we have the MLE of $\left(\hat{\omega },\hat{a},\hat{b}\right)=\left(1.969,0.152,0.664\right)$ and the MDPDE of $\left(\hat{\omega },\hat{a},\hat{b}\right)$ = (1.213,0.144,0.472) when α=0.1 is used. The significant difference between the two estimates is seemingly due to the presence of outliers. Using $Y_{t},t=1,\ldots ,150$ as a training sample and viewing $Y_{t}$, t≥151 as sequentially observed testing data, we implement the monitoring process ${\hat{T}}_{n}^{cusum}$ with α=0,0.1 to detect a parameter change. Subsequently, an anomaly is detected when t=180 for α=0 (blue vertical line) and t=197 for α=0.1 (red vertical line), which indicates that the monitoring process based on the MLE is more sensitive to relatively smaller outliers lying around t=180, while that based on MDPDE is more robust to those outliers and detects a more significant change around t=197, ignoring smaller ones. Obviously, we can see from Figure 3 that $Y_{t}$ has a pattern with more fluctuations after t=180. Our finding affirms the adequacy of the MDPDE-based monitoring process in the presence of outliers.

## 6. Concluding Remarks

In this work, we studied the robust on-line monitoring process based on MDPDE for detecting a parameter change in INGARCH models. For this task, we adopted the CUSUM process based on the score functions, which were originally constructed for obtaining the MDPDE. Our simulation study and real data analysis confirmed the validity of the proposed method. Here, we focused on the monitoring process within the framework of Gombay and Serban [30] and Huh, Kim and Lee [31]. However, one can also consider a different monitoring scheme, for example as in Na, Lee and Lee [44], and conduct a comparison study, which is left as our future project.

## Figures and Tables

**Table 1:** Empirical sizes and powers in Case 1 for the Poisson INGARCH(1,1) model when no outliers exist with $\theta_{0}=\left(2,0.1,0.2\right)$.

	α	*n*	τ	δ:	0	0.25	0.5	0.75	1
${\hat{T}}_{n,0}^{min}$	0	500	250		0.035	0.541	1	1	1
${\hat{T}}_{n}^{min}$	0	500	250		0.036	0.428	1	1	1
${\hat{T}}_{n}^{cusum}$	0	500	250		0.048	0.997	1	1	1
${\hat{T}}_{n,0}^{min}$	0	1000	500		0.042	0.791	1	1	1
${\hat{T}}_{n}^{min}$	0	1000	500		0.049	0.682	1	1	1
${\hat{T}}_{n}^{cusum}$	0	1000	500		0.052	1	1	1	1
${\hat{T}}_{n,0}^{min}$	0.1	500	250		0.035	0.523	1	1	1
${\hat{T}}_{n}^{min}$	0.1	500	250		0.036	0.398	1	1	1
${\hat{T}}_{n}^{cusum}$	0.1	500	250		0.043	0.995	1	1	1
${\hat{T}}_{n,0}^{min}$	0.1	1000	500		0.042	0.78	1	1	1
${\hat{T}}_{n}^{min}$	0.1	1000	500		0.051	0.642	1	1	1
${\hat{T}}_{n}^{cusum}$	0.1	1000	500		0.056	1	1	1	1
${\hat{T}}_{n,0}^{min}$	0.2	500	250		0.035	0.493	1	1	1
${\hat{T}}_{n}^{min}$	0.2	500	250		0.038	0.361	1	1	1
${\hat{T}}_{n}^{cusum}$	0.2	500	250		0.041	0.994	1	1	1
${\hat{T}}_{n,0}^{min}$	0.2	1000	500		0.04	0.757	1	1	1
${\hat{T}}_{n}^{min}$	0.2	1000	500		0.048	0.589	1	1	1
${\hat{T}}_{n}^{cusum}$	0.2	1000	500		0.066	1	1	1	1
${\hat{T}}_{n,0}^{min}$	0.3	500	250		0.035	0.465	1	1	1
${\hat{T}}_{n}^{min}$	0.3	500	250		0.042	0.332	1	1	1
${\hat{T}}_{n}^{cusum}$	0.3	500	250		0.036	0.992	1	1	1
${\hat{T}}_{n,0}^{min}$	0.3	1000	500		0.034	0.718	1	1	1
${\hat{T}}_{n}^{min}$	0.3	1000	500		0.047	0.551	1	1	1
${\hat{T}}_{n}^{cusum}$	0.3	1000	500		0.064	1	1	1	1

**Table 2:** Empirical sizes and powers in Case 2 for the Poisson INGARCH(1,1) model when no outliers exist with $\theta_{0}=\left(2,0.6,0.2\right)$.

	α	*n*	τ	δ:	0	−1/5	−1/3	−3/7	−1/2
${\hat{T}}_{n,0}^{min}$	0	500	250		0.05	0.983	1	1	1
${\hat{T}}_{n}^{min}$	0	500	250		0.06	0.86	0.999	1	1
${\hat{T}}_{n}^{cusum}$	0	500	250		0.049	0.893	0.999	1	1
${\hat{T}}_{n,0}^{min}$	0	1000	500		0.052	1	1	1	1
${\hat{T}}_{n}^{min}$	0	1000	500		0.053	0.98	1	1	1
${\hat{T}}_{n}^{cusum}$	0	1000	500		0.059	0.997	1	1	1
${\hat{T}}_{n,0}^{min}$	0.1	500	250		0.047	0.984	1	1	1
${\hat{T}}_{n}^{min}$	0.1	500	250		0.058	0.871	1	1	1
${\hat{T}}_{n}^{cusum}$	0.1	500	250		0.046	0.9	1	1	1
${\hat{T}}_{n,0}^{min}$	0.1	1000	500		0.048	1	1	1	1
${\hat{T}}_{n}^{min}$	0.1	1000	500		0.041	0.977	1	1	1
${\hat{T}}_{n}^{cusum}$	0.1	1000	500		0.051	0.996	1	1	1
${\hat{T}}_{n,0}^{min}$	0.2	500	250		0.045	0.986	1	1	1
${\hat{T}}_{n}^{min}$	0.2	500	250		0.05	0.852	0.999	1	1
${\hat{T}}_{n}^{cusum}$	0.2	500	250		0.043	0.904	1	1	1
${\hat{T}}_{n,0}^{min}$	0.2	1000	500		0.052	1	1	1	1
${\hat{T}}_{n}^{min}$	0.2	1000	500		0.04	0.973	1	1	1
${\hat{T}}_{n}^{cusum}$	0.2	1000	500		0.054	0.997	1	1	1
${\hat{T}}_{n,0}^{min}$	0.3	500	250		0.04	0.985	1	1	1
${\hat{T}}_{n}^{min}$	0.3	500	250		0.043	0.845	0.999	1	1
${\hat{T}}_{n}^{cusum}$	0.3	500	250		0.048	0.912	1	1	1
${\hat{T}}_{n,0}^{min}$	0.3	1000	500		0.05	1	1	1	1
${\hat{T}}_{n}^{min}$	0.3	1000	500		0.052	0.978	1	1	1
${\hat{T}}_{n}^{cusum}$	0.3	1000	500		0.053	0.996	1	1	1

**Table 3:** Empirical sizes and powers in Case 3 for the Poisson INGARCH(1,1) model when no outliers exist with $\theta_{0}=\left(2,0.3,0.3\right)$.

	α	*n*	τ	δ:	0	0.25	0.5	0.75	1
${\hat{T}}_{n,0}^{min}$	0	500	250		0.046	0.309	0.999	1	1
${\hat{T}}_{n}^{min}$	0	500	250		0.043	0.216	0.993	1	1
${\hat{T}}_{n}^{cusum}$	0	500	250		0.047	0.685	1	1	1
${\hat{T}}_{n,0}^{min}$	0	1000	500		0.039	0.473	1	1	1
${\hat{T}}_{n}^{min}$	0	1000	500		0.041	0.337	1	1	1
${\hat{T}}_{n}^{cusum}$	0	1000	500		0.057	0.969	1	1	1
${\hat{T}}_{n,0}^{min}$	0.1	500	250		0.044	0.292	0.999	1	1
${\hat{T}}_{n}^{min}$	0.1	500	250		0.046	0.208	0.992	1	1
${\hat{T}}_{n}^{cusum}$	0.1	500	250		0.054	0.696	1	1	1
${\hat{T}}_{n,0}^{min}$	0.1	1000	500		0.046	0.458	1	1	1
${\hat{T}}_{n}^{min}$	0.1	1000	500		0.047	0.314	1	1	1
${\hat{T}}_{n}^{cusum}$	0.1	1000	500		0.062	0.965	1	1	1
${\hat{T}}_{n,0}^{min}$	0.2	500	250		0.046	0.266	0.998	1	1
${\hat{T}}_{n}^{min}$	0.2	500	250		0.05	0.192	0.99	1	1
${\hat{T}}_{n}^{cusum}$	0.2	500	250		0.048	0.696	1	1	1
${\hat{T}}_{n,0}^{min}$	0.2	1000	500		0.044	0.44	1	1	1
${\hat{T}}_{n}^{min}$	0.2	1000	500		0.042	0.287	1	1	1
${\hat{T}}_{n}^{cusum}$	0.2	1000	500		0.067	0.962	1	1	1
${\hat{T}}_{n,0}^{min}$	0.3	500	250		0.041	0.244	0.998	1	1
${\hat{T}}_{n}^{min}$	0.3	500	250		0.051	0.179	0.986	1	1
${\hat{T}}_{n}^{cusum}$	0.3	500	250		0.051	0.669	1	1	1
${\hat{T}}_{n,0}^{min}$	0.3	1000	500		0.04	0.412	1	1	1
${\hat{T}}_{n}^{min}$	0.3	1000	500		0.045	0.267	1	1	1
${\hat{T}}_{n}^{cusum}$	0.3	1000	500		0.055	0.956	1	1	1

**Table 4:** Empirical sizes and powers in Case 4 for the Poisson INGARCH(1,1) model when no outliers exist with $\theta_{0}=\left(1,0.4,0.4\right)$.

	α	*n*	τ	δ:	0	−1/5	−1/3	−3/7	−1/2
${\hat{T}}_{n,0}^{min}$	0	500	250		0.044	0.687	0.991	1	1
${\hat{T}}_{n}^{min}$	0	500	250		0.049	0.345	0.75	0.941	0.986
${\hat{T}}_{n}^{cusum}$	0	500	250		0.058	0.364	0.828	0.957	0.991
${\hat{T}}_{n,0}^{min}$	0	1000	500		0.038	0.946	1	1	1
${\hat{T}}_{n}^{min}$	0	1000	500		0.039	0.626	0.969	1	1
${\hat{T}}_{n}^{cusum}$	0	1000	500		0.058	0.796	0.998	1	1
${\hat{T}}_{n,0}^{min}$	0.1	500	250		0.044	0.688	0.99	1	1
${\hat{T}}_{n}^{min}$	0.1	500	250		0.054	0.349	0.752	0.938	0.985
${\hat{T}}_{n}^{cusum}$	0.1	500	250		0.06	0.376	0.841	0.964	0.993
${\hat{T}}_{n,0}^{min}$	0.1	1000	500		0.042	0.945	1	1	1
${\hat{T}}_{n}^{min}$	0.1	1000	500		0.042	0.616	0.966	0.999	1
${\hat{T}}_{n}^{cusum}$	0.1	1000	500		0.053	0.782	0.997	1	1
${\hat{T}}_{n,0}^{min}$	0.2	500	250		0.047	0.686	0.989	0.999	1
${\hat{T}}_{n}^{min}$	0.2	500	250		0.056	0.357	0.757	0.939	0.986
${\hat{T}}_{n}^{cusum}$	0.2	500	250		0.056	0.378	0.832	0.965	0.991
${\hat{T}}_{n,0}^{min}$	0.2	1000	500		0.042	0.94	1	1	1
${\hat{T}}_{n}^{min}$	0.2	1000	500		0.039	0.597	0.965	0.999	1
${\hat{T}}_{n}^{cusum}$	0.2	1000	500		0.059	0.793	0.997	1	1
${\hat{T}}_{n,0}^{min}$	0.3	500	250		0.049	0.677	0.985	0.999	1
${\hat{T}}_{n}^{min}$	0.3	500	250		0.048	0.321	0.721	0.917	0.977
${\hat{T}}_{n}^{cusum}$	0.3	500	250		0.054	0.381	0.831	0.963	0.991
${\hat{T}}_{n,0}^{min}$	0.3	1000	500		0.043	0.931	1	1	1
${\hat{T}}_{n}^{min}$	0.3	1000	500		0.047	0.606	0.962	0.999	1
${\hat{T}}_{n}^{cusum}$	0.3	1000	500		0.064	0.792	0.997	1	1

**Table 5:** Empirical sizes and powers in Case 1 for the Poisson INGARCH(1,1) model when no outliers exist with $\theta_{0}=\left(2,0.1,0.2\right)$.

	α	*n*	τ	δ:	0	0.25	0.5	0.75	1
${\hat{T}}_{n,0}^{min}$	0	500	125		0.035	0.759	1	1	1
${\hat{T}}_{n}^{min}$	0	500	125		0.036	0.636	1	1	1
${\hat{T}}_{n}^{cusum}$	0	500	125		0.048	0.983	1	1	1
${\hat{T}}_{n,0}^{min}$	0	1000	250		0.042	0.936	1	1	1
${\hat{T}}_{n}^{min}$	0	1000	250		0.049	0.874	1	1	1
${\hat{T}}_{n}^{cusum}$	0	1000	250		0.052	1	1	1	1
${\hat{T}}_{n,0}^{min}$	0.1	500	125		0.035	0.739	1	1	1
${\hat{T}}_{n}^{min}$	0.1	500	125		0.036	0.606	1	1	1
${\hat{T}}_{n}^{cusum}$	0.1	500	125		0.043	0.981	1	1	1
${\hat{T}}_{n,0}^{min}$	0.1	1000	250		0.042	0.938	1	1	1
${\hat{T}}_{n}^{min}$	0.1	1000	250		0.051	0.861	1	1	1
${\hat{T}}_{n}^{cusum}$	0.1	1000	250		0.056	1	1	1	1
${\hat{T}}_{n,0}^{min}$	0.2	500	125		0.035	0.716	1	1	1
${\hat{T}}_{n}^{min}$	0.2	500	125		0.038	0.57	1	1	1
${\hat{T}}_{n}^{cusum}$	0.2	500	125		0.041	0.981	1	1	1
${\hat{T}}_{n,0}^{min}$	0.2	1000	250		0.04	0.936	1	1	1
${\hat{T}}_{n}^{min}$	0.2	1000	250		0.048	0.842	1	1	1
${\hat{T}}_{n}^{cusum}$	0.2	1000	250		0.066	1	1	1	1
${\hat{T}}_{n,0}^{min}$	0.3	500	125		0.035	0.693	1	1	1
${\hat{T}}_{n}^{min}$	0.3	500	125		0.042	0.542	1	1	1
${\hat{T}}_{n}^{cusum}$	0.3	500	125		0.036	0.976	1	1	1
${\hat{T}}_{n,0}^{min}$	0.3	1000	250		0.034	0.93	1	1	1
${\hat{T}}_{n}^{min}$	0.3	1000	250		0.047	0.828	1	1	1
${\hat{T}}_{n}^{cusum}$	0.3	1000	250		0.064	1	1	1	1

**Table 6:** Empirical sizes and powers Case 2 for the Poisson INGARCH(1,1) model when no outliers exist with $\theta_{0}=\left(2,0.6,0.2\right)$.

	α	*n*	τ	δ:	0	−1/5	−1/3	−3/7	−1/2
${\hat{T}}_{n,0}^{min}$	0	500	125		0.05	0.999	1	1	1
${\hat{T}}_{n}^{min}$	0	500	125		0.06	0.969	1	1	1
${\hat{T}}_{n}^{cusum}$	0	500	125		0.049	0.844	1	1	1
${\hat{T}}_{n,0}^{min}$	0	1000	250		0.052	1	1	1	1
${\hat{T}}_{n}^{min}$	0	1000	250		0.053	1	1	1	1
${\hat{T}}_{n}^{cusum}$	0	1000	250		0.059	0.988	1	1	1
${\hat{T}}_{n,0}^{min}$	0.1	500	125		0.047	1	1	1	1
${\hat{T}}_{n}^{min}$	0.1	500	125		0.058	0.971	1	1	1
${\hat{T}}_{n}^{cusum}$	0.1	500	125		0.046	0.85	1	1	1
${\hat{T}}_{n,0}^{min}$	0.1	1000	250		0.048	1	1	1	1
${\hat{T}}_{n}^{min}$	0.1	1000	250		0.041	1	1	1	1
${\hat{T}}_{n}^{cusum}$	0.1	1000	250		0.051	0.988	1	1	1
${\hat{T}}_{n,0}^{min}$	0.2	500	125		0.045	1	1	1	1
${\hat{T}}_{n}^{min}$	0.2	500	125		0.05	0.967	1	1	1
${\hat{T}}_{n}^{cusum}$	0.2	500	125		0.043	0.845	1	1	1
${\hat{T}}_{n,0}^{min}$	0.2	1000	250		0.052	1	1	1	1
${\hat{T}}_{n}^{min}$	0.2	1000	250		0.04	1	1	1	1
${\hat{T}}_{n}^{cusum}$	0.2	1000	250		0.054	0.991	1	1	1
${\hat{T}}_{n,0}^{min}$	0.3	500	125		0.04	1	1	1	1
${\hat{T}}_{n}^{min}$	0.3	500	125		0.043	0.962	1	1	1
${\hat{T}}_{n}^{cusum}$	0.3	500	125		0.048	0.863	1	1	1
${\hat{T}}_{n,0}^{min}$	0.3	1000	250		0.05	1	1	1	1
${\hat{T}}_{n}^{min}$	0.3	1000	250		0.052	1	1	1	1
${\hat{T}}_{n}^{cusum}$	0.3	1000	250		0.053	0.986	1	1	1

**Table 7:** Empirical sizes and powers Case 3 for the Poisson INGARCH(1,1) model when no outliers exist with $\theta_{0}=\left(2,0.3,0.3\right)$.

	α	*n*	τ	δ:	0	0.25	0.5	0.75	1
${\hat{T}}_{n,0}^{min}$	0	500	125		0.046	0.488	1	1	1
${\hat{T}}_{n}^{min}$	0	500	125		0.043	0.33	0.999	1	1
${\hat{T}}_{n}^{cusum}$	0	500	125		0.047	0.614	1	1	1
${\hat{T}}_{n,0}^{min}$	0	1000	250		0.039	0.716	1	1	1
${\hat{T}}_{n}^{min}$	0	1000	250		0.041	0.554	1	1	1
${\hat{T}}_{n}^{cusum}$	0	1000	250		0.057	0.916	1	1	1
${\hat{T}}_{n,0}^{min}$	0.1	500	125		0.044	0.455	1	1	1
${\hat{T}}_{n}^{min}$	0.1	500	125		0.046	0.314	0.999	1	1
${\hat{T}}_{n}^{cusum}$	0.1	500	125		0.054	0.614	1	1	1
${\hat{T}}_{n,0}^{min}$	0.1	1000	250		0.046	0.706	1	1	1
${\hat{T}}_{n}^{min}$	0.1	1000	250		0.047	0.531	1	1	1
${\hat{T}}_{n}^{cusum}$	0.1	1000	250		0.062	0.914	1	1	1
${\hat{T}}_{n,0}^{min}$	0.2	500	125		0.046	0.434	1	1	1
${\hat{T}}_{n}^{min}$	0.2	500	125		0.05	0.295	0.999	1	1
${\hat{T}}_{n}^{cusum}$	0.2	500	125		0.048	0.601	0.999	1	1
${\hat{T}}_{n,0}^{min}$	0.2	1000	250		0.044	0.701	1	1	1
${\hat{T}}_{n}^{min}$	0.2	1000	250		0.042	0.505	1	1	1
${\hat{T}}_{n}^{cusum}$	0.2	1000	250		0.067	0.901	1	1	1
${\hat{T}}_{n,0}^{min}$	0.3	500	125		0.041	0.416	1	1	1
${\hat{T}}_{n}^{min}$	0.3	500	125		0.051	0.283	0.999	1	1
${\hat{T}}_{n}^{cusum}$	0.3	500	125		0.051	0.573	0.999	1	1
${\hat{T}}_{n,0}^{min}$	0.3	1000	250		0.04	0.684	1	1	1
${\hat{T}}_{n}^{min}$	0.3	1000	250		0.045	0.485	1	1	1
${\hat{T}}_{n}^{cusum}$	0.3	1000	250		0.055	0.869	1	1	1

**Table 8:** Empirical sizes and powers in Case 4 for the Poisson INGARCH(1,1) model when no outliers exist with $\theta_{0}=\left(1,0.4,0.4\right)$.

	α	*n*	τ	δ:	0	−1/5	−1/3	−3/7	−1/2
${\hat{T}}_{n,0}^{min}$	0	500	125		0.044	0.958	1	1	1
${\hat{T}}_{n}^{min}$	0	500	125		0.049	0.559	0.937	0.995	0.999
${\hat{T}}_{n}^{cusum}$	0	500	125		0.058	0.242	0.636	0.869	0.943
${\hat{T}}_{n,0}^{min}$	0	1000	250		0.038	0.998	1	1	1
${\hat{T}}_{n}^{min}$	0	1000	250		0.039	0.887	1	1	1
${\hat{T}}_{n}^{cusum}$	0	1000	250		0.058	0.543	0.961	0.998	1
${\hat{T}}_{n,0}^{min}$	0.1	500	125		0.044	0.955	1	1	1
${\hat{T}}_{n}^{min}$	0.1	500	125		0.054	0.565	0.937	0.994	0.999
${\hat{T}}_{n}^{cusum}$	0.1	500	125		0.06	0.283	0.667	0.881	0.953
${\hat{T}}_{n,0}^{min}$	0.1	1000	250		0.042	0.999	1	1	1
${\hat{T}}_{n}^{min}$	0.1	1000	250		0.042	0.883	1	1	1
${\hat{T}}_{n}^{cusum}$	0.1	1000	250		0.053	0.542	0.96	0.998	1
${\hat{T}}_{n,0}^{min}$	0.2	500	125		0.047	0.95	1	1	1
${\hat{T}}_{n}^{min}$	0.2	500	125		0.056	0.574	0.941	0.992	0.999
${\hat{T}}_{n}^{cusum}$	0.2	500	125		0.056	0.291	0.669	0.88	0.951
${\hat{T}}_{n,0}^{min}$	0.2	1000	250		0.042	0.999	1	1	1
${\hat{T}}_{n}^{min}$	0.2	1000	250		0.039	0.873	0.997	1	1
${\hat{T}}_{n}^{cusum}$	0.2	1000	250		0.059	0.56	0.965	0.999	1
${\hat{T}}_{n,0}^{min}$	0.3	500	125		0.049	0.945	1	1	1
${\hat{T}}_{n}^{min}$	0.3	500	125		0.048	0.535	0.931	0.987	0.997
${\hat{T}}_{n}^{cusum}$	0.3	500	125		0.054	0.294	0.662	0.873	0.95
${\hat{T}}_{n,0}^{min}$	0.3	1000	250		0.043	0.999	1	1	1
${\hat{T}}_{n}^{min}$	0.3	1000	250		0.047	0.885	0.996	1	1
${\hat{T}}_{n}^{cusum}$	0.3	1000	250		0.064	0.569	0.967	0.998	1

**Table 9:** Empirical sizes and powers in Case 1 for the Poisson INGARCH(1,1) model when $\theta_{0}=\left(2,0.1,0.2\right)$, p=0.1 and λ=10.

	α	*n*	τ	δ:	0	0.25	0.5	0.75	1
${\hat{T}}_{n}^{min}$	0	500	250		0.065	0.058	0.145	0.8	0.958
${\hat{T}}_{n}^{cusum}$	0	500	250		0.048	0.047	0.066	0.512	0.997
${\hat{T}}_{n}^{min}$	0	1000	500		0.061	0.058	0.367	0.958	0.991
${\hat{T}}_{n}^{cusum}$	0	1000	500		0.053	0.053	0.095	0.978	1
${\hat{T}}_{n}^{min}$	0.1	500	250		0.042	0.039	0.23	0.891	0.962
${\hat{T}}_{n}^{cusum}$	0.1	500	250		0.035	0.037	0.122	0.897	1
${\hat{T}}_{n}^{min}$	0.1	1000	500		0.056	0.046	0.653	0.979	0.995
${\hat{T}}_{n}^{cusum}$	0.1	1000	500		0.053	0.054	0.963	1	1
${\hat{T}}_{n}^{min}$	0.2	500	250		0.036	0.032	0.162	0.842	0.951
${\hat{T}}_{n}^{cusum}$	0.2	500	250		0.035	0.036	0.111	0.804	1
${\hat{T}}_{n}^{min}$	0.2	1000	500		0.026	0.025	0.454	0.976	0.993
${\hat{T}}_{n}^{cusum}$	0.2	1000	500		0.023	0.023	0.514	1	1
${\hat{T}}_{n}^{min}$	0.3	500	250		0.032	0.034	0.201	0.855	0.95
${\hat{T}}_{n}^{cusum}$	0.3	500	250		0.032	0.032	0.114	0.771	0.979
${\hat{T}}_{n}^{min}$	0.3	1000	500		0.024	0.02	0.485	0.973	0.991
${\hat{T}}_{n}^{cusum}$	0.3	1000	500		0.021	0.021	0.284	0.999	1

**Table 10:** Empirical sizes and powers in Case 2 for the Poisson INGARCH(1,1) model when $\theta_{0}=\left(2,0.6,0.2\right)$, p=0.1 and λ=30.

	α	*n*	τ	δ:	0	0.25	0.5	0.75	1
${\hat{T}}_{n}^{min}$	0	500	250		0.08	0.975	1	1	1
${\hat{T}}_{n}^{cusum}$	0	500	250		0.065	0.11	0.194	0.329	0.456
${\hat{T}}_{n}^{min}$	0	1000	500		0.055	1	1	1	1
${\hat{T}}_{n}^{cusum}$	0	1000	500		0.05	0.203	0.594	0.795	0.902
${\hat{T}}_{n}^{min}$	0.1	500	250		0.057	0.935	0.999	1	1
${\hat{T}}_{n}^{cusum}$	0.1	500	250		0.062	0.169	0.666	0.927	0.993
${\hat{T}}_{n}^{min}$	0.1	1000	500		0.091	0.999	1	1	1
${\hat{T}}_{n}^{cusum}$	0.1	1000	500		0.052	0.615	1	1	1
${\hat{T}}_{n}^{min}$	0.2	500	250		0.054	0.875	0.998	0.999	1
${\hat{T}}_{n}^{cusum}$	0.2	500	250		0.043	0.069	0.309	0.663	0.784
${\hat{T}}_{n}^{min}$	0.2	1000	500		0.135	0.993	1	1	1
${\hat{T}}_{n}^{cusum}$	0.2	1000	500		0.046	0.569	0.999	1	1
${\hat{T}}_{n}^{min}$	0.3	500	250		0.063	0.896	0.998	0.999	1
${\hat{T}}_{n}^{cusum}$	0.3	500	250		0.046	0.086	0.455	0.763	0.853
${\hat{T}}_{n}^{min}$	0.3	1000	500		0.159	0.992	1	1	1
${\hat{T}}_{n}^{cusum}$	0.3	1000	500		0.047	0.675	0.999	1	1

**Table 11:** Empirical sizes and powers in Case 3 for the Poisson INGARCH(1,1) model when $\theta_{0}=\left(2,0.3,0.3\right)$, p=0.1 and λ=30.

	α	*n*	τ	δ:	0	0.25	0.5	0.75	1
${\hat{T}}_{n}^{min}$	0	500	250		0.074	0.118	0.069	0.127	0.885
${\hat{T}}_{n}^{cusum}$	0	500	250		0.062	0.064	0.06	0.068	0.777
${\hat{T}}_{n}^{min}$	0	1000	500		0.062	0.213	0.058	0.257	0.935
${\hat{T}}_{n}^{cusum}$	0	1000	500		0.049	0.05	0.049	0.057	0.992
${\hat{T}}_{n}^{min}$	0.1	500	250		0.036	0.033	0.041	0.516	0.914
${\hat{T}}_{n}^{cusum}$	0.1	500	250		0.037	0.037	0.04	0.268	0.961
${\hat{T}}_{n}^{min}$	0.1	1000	500		0.029	0.026	0.03	0.824	0.963
${\hat{T}}_{n}^{cusum}$	0.1	1000	500		0.023	0.023	0.025	0.859	1
${\hat{T}}_{n}^{min}$	0.2	500	250		0.038	0.034	0.038	0.487	0.865
${\hat{T}}_{n}^{cusum}$	0.2	500	250		0.04	0.042	0.046	0.321	0.612
${\hat{T}}_{n}^{min}$	0.2	1000	500		0.019	0.017	0.018	0.725	0.922
${\hat{T}}_{n}^{cusum}$	0.2	1000	500		0.015	0.015	0.015	0.244	0.616
${\hat{T}}_{n}^{min}$	0.3	500	250		0.035	0.032	0.036	0.351	0.661
${\hat{T}}_{n}^{cusum}$	0.3	500	250		0.039	0.039	0.042	0.13	0.211
${\hat{T}}_{n}^{min}$	0.3	1000	500		0.02	0.016	0.017	0.684	0.893
${\hat{T}}_{n}^{cusum}$	0.3	1000	500		0.012	0.012	0.012	0.085	0.161

**Table 12:** Empirical sizes and powers in Case 4 for the Poisson INGARCH(1,1) model when $\theta_{0}=\left(1,0.4,0.4\right)$, p=0.1 and λ=30.

	α	*n*	τ	δ:	0	0.25	0.5	0.75	1
${\hat{T}}_{n}^{min}$	0	500	250		0.05	0.796	0.958	0.989	0.996
${\hat{T}}_{n}^{cusum}$	0	500	250		0.048	0.078	0.118	0.173	0.219
${\hat{T}}_{n}^{min}$	0	1000	500		0.032	1	1	1	1
${\hat{T}}_{n}^{cusum}$	0	1000	500		0.043	0.613	0.874	0.931	0.957
${\hat{T}}_{n}^{min}$	0.1	500	250		0.085	0.712	0.97	0.997	1
${\hat{T}}_{n}^{cusum}$	0.1	500	250		0.04	0.065	0.243	0.466	0.647
${\hat{T}}_{n}^{min}$	0.1	1000	500		0.242	0.978	0.999	1	1
${\hat{T}}_{n}^{cusum}$	0.1	1000	500		0.069	0.916	0.999	1	1
${\hat{T}}_{n}^{min}$	0.2	500	250		0.078	0.677	0.96	0.995	0.999
${\hat{T}}_{n}^{cusum}$	0.2	500	250		0.032	0.069	0.284	0.535	0.735
${\hat{T}}_{n}^{min}$	0.2	1000	500		0.229	0.965	0.999	1	1
${\hat{T}}_{n}^{cusum}$	0.2	1000	500		0.047	0.836	0.999	1	1
${\hat{T}}_{n}^{min}$	0.3	500	250		0.06	0.642	0.947	0.993	0.999
${\hat{T}}_{n}^{cusum}$	0.3	500	250		0.027	0.08	0.332	0.621	0.807
${\hat{T}}_{n}^{min}$	0.3	1000	500		0.201	0.962	0.999	1	1
${\hat{T}}_{n}^{cusum}$	0.3	1000	500		0.027	0.749	0.999	1	1

## Abbreviations

The following abbreviations are used in this manuscript:

CUSUM	cumulative sum
INGARCH	integer-valued generalized autoregressive conditionally heteroscedastic
INAR	integer-valued autoregressive
MDPDE	minimum density power divergence etimator
MLE	maximum likelihood estimator
SPC	statistical process control

## Appendix A

**Proof of** **Theorem 1.** We first verify that ${\hat{T}}_{n}^{max}$ converges to *T* in distribution; the cases of ${\hat{T}}_{n,0}^{min}$, ${\hat{T}}_{n,0}^{max}$, and ${\hat{T}}_{n}^{min}$ can be similarly handled and the proofs for these are omitted. As ${\hat{\theta }}_{\alpha ,m}$ converges to $\theta_{0}$ and

$$\begin{array}{c} E\left(\underset{\theta \in \Theta }{\sup}∥\frac{\partial^{2}l_{\alpha ,t}\left(\theta \right)}{\partial \theta \partial \theta^{T}}-\frac{\partial^{2}l_{\alpha ,t}\left(\theta_{0}\right)}{\partial \theta \partial \theta^{T}}∥\right)<\infty , \end{array}$$

we have that for any sequence $\theta_{n}^{*}$ converging to $\theta_{0}$ a.s.,

(A1) $$\begin{array}{c} \frac{1}{n}\sum_{t=1}^{n}\frac{\partial^{2}l_{\alpha ,t}\left(\theta_{n}^{*}\right)}{\partial \theta \partial \theta^{T}}\to -J_{\alpha } \end{array}$$

in probability. Then, using the mean value theorem and ergodicity, owing to (A1), we have

(A2) $$\begin{array}{ccc} & & \max_{1\leq k\leq n}\max_{1\leq j\leq k}||\frac{1}{\sqrt{n}}\sum_{t=1}^{j}\frac{\partial l_{\alpha ,t}\left({\hat{\theta }}_{\alpha ,m}\right)}{\partial \theta }-\frac{1}{\sqrt{n}}\sum_{t=1}^{k}\frac{\partial l_{\alpha ,t}\left({\hat{\theta }}_{\alpha ,m}\right)}{\partial \theta }\\ & & \;\;\;\;-\left\{\frac{1}{\sqrt{n}}\sum_{t=1}^{j}\frac{\partial l_{\alpha ,t}\left(\theta_{0}\right)}{\partial \theta }-\frac{1}{\sqrt{n}}\sum_{t=1}^{k}\frac{\partial l_{\alpha ,t}\left(\theta_{0}\right)}{\partial \theta }\right\}||\\ & & \leq \sqrt{m}\left|\right|{\hat{\theta }}_{\alpha ,m}-\theta_{0}\left|\right|\sqrt{\frac{n}{m}}\max_{1\leq j,k\leq n}\left|\left|\left(\frac{j}{n}\right)\frac{1}{j}\sum_{t=1}^{j}\frac{\partial^{2}l_{\alpha ,t}\left(\theta_{n}^{*}\right)}{\partial \theta \partial \theta^{T}}-\left(\frac{k}{n}\right)\frac{1}{k}\sum_{t=1}^{k}\frac{\partial^{2}l_{\alpha ,t}\left(\theta_{n}^{**}\right)}{\partial \theta \partial \theta^{T}}\right|\right|\\ & & =o_{P}\left(1\right), \end{array}$$

where ${\hat{\theta }}_{n}^{*}$ and ${\hat{\theta }}_{n}^{**}$ are intermediate points between $\theta_{0}$ and ${\hat{\theta }}_{\alpha ,m}$. Hence, since ${\hat{K}}_{\alpha ,m}$ is a consistent estimator of $K_{\alpha }$ (Lemma A5 of Kim and Lee [36]) and

(A3) $$\begin{array}{c} \underset{\theta \in \Theta }{\sup}\max_{1\leq k\leq n}\left|\left|\frac{1}{\sqrt{n}}\sum_{t=1}^{k}\frac{\partial {\tilde{l}}_{\alpha ,t}\left(\theta \right)}{\partial \theta }-\frac{1}{\sqrt{n}}\sum_{t=1}^{k}\frac{\partial l_{\alpha ,t}\left(\theta \right)}{\partial \theta }\right|\right|=o_{P}\left(1\right) \end{array}$$

(Lemma 6 of Kim and Lee, 2019), we get ${\hat{T}}_{n}^{max}-T_{n}^{max}=o_{P}\left(1\right)$ and ${\hat{T}}_{n}^{max}$ converges to *T* in distribution owing to (9). Next, we deal with ${\hat{T}}_{n}^{cusum}$. The case of ${\hat{T}}_{n,0}^{cusum}$ can be similarly handled. Similarly to (A2), we can see that

(A4) $$\begin{array}{ccc} & & \max_{1\leq k\leq n}||\frac{1}{\sqrt{n}}\sum_{t=1}^{k}\frac{\partial l_{\alpha ,t}\left({\hat{\theta }}_{\alpha ,m}\right)}{\partial \theta }-\frac{1}{\sqrt{n}}\left(\frac{k}{n}\right)\sum_{t=1}^{n}\frac{\partial l_{\alpha ,t}\left({\hat{\theta }}_{\alpha ,m}\right)}{\partial \theta }\\ & & -\frac{1}{\sqrt{n}}\sum_{t=1}^{k}\frac{\partial l_{\alpha ,t}\left(\theta_{0}\right)}{\partial \theta }-\frac{1}{\sqrt{n}}\left(\frac{k}{n}\right)\sum_{t=1}^{n}\frac{\partial l_{\alpha ,t}\left(\theta_{0}\right)}{\partial \theta }||=o_{P}\left(1\right). \end{array}$$

Then, using the arguments as in (A3) and (A4), we can see that

$$\begin{array}{c} \max_{1\leq k\leq n}\frac{1}{\sqrt{n}}\left|\left|{\hat{W}}_{k}-\left(\frac{k}{n}\right){\hat{W}}_{k}-W_{k}+\left(\frac{k}{n}\right)W_{k}\right|\right|=o_{P}\left(1\right), \end{array}$$

which implies ${\hat{T}}_{n}^{cusum}-T_{n}^{cusum}=o_{P}\left(1\right)$ and ${\hat{T}}_{n}^{cusum}\overset{d}{\to }T^{{}^{\prime }}$ holds owing to (9). This completes the proof. □
